# Sports Supplement Consumption in 316 Federated Female Road Cyclists

**DOI:** 10.3390/nu16152563

**Published:** 2024-08-04

**Authors:** Jesús García-Durán, José Antonio González-Jurado, Antonio Jesús Sánchez-Oliver

**Affiliations:** 1 Faculty of Sports Science, Pablo de Olavide University, 41013 Seville, Spain; 2 Research Center on Physical and Sports Performance, Pablo de Olavide University, 41013 Seville, Spain; 3 Departamento de Motricidad Humana y Rendimiento Deportivo, Universidad de Sevilla, 41013 Sevilla, Spain

**Keywords:** sports supplements, cycling, sport nutrition, ergogenic aids, performance, doping

## Abstract

Although the extensive use of sports supplements (SSs) is prevalent among cyclists, this area has been poorly explored; in fact, no studies have been conducted on this topic regarding women cyclists to date. This descriptive, cross-sectional study, which included 316 federated female road cyclists, aimed to analyze SS consumption patterns in relation to scientific evidence and various categories. SSs were categorized according to the groups and subgroups established by the Australian Sport Institute (AIS, 2023) based on the level of evidence supporting their use. The analysis found that 85.1% of the female road cyclists surveyed used SSs, with an average consumption of 7 ± 6 supplements per individual. Pharmacies (60.8%), dietitian-nutritionists (58.9%), and health status (60.1%) were the primary purchase location, source of information, and reason for use, respectively. The most frequently consumed supplements were sports bars (77.5%), sports gels (61.4%), and caffeine (49.1%). Significantly, 80% of the ten most commonly used supplements were from the group with the highest evidence level, as classified by the AIS, with an average intake of 5 ± 3 supplements per cyclist. In summary, the use of SSs is prevalent among female road cyclists, with reliable sources for both purchasing and obtaining advice on supplements.

## 1. Introduction

The number of federative licenses in women’s cycling in Spain has increased in recent years. Contributing factors to this rise include geographical location, climate, and the promotion of the sport (Spanish Cycling Federation, 2023) [1]. Throughout the year, various cycling competitions take place, including road, mountain, and recently, “gravel” events. These competitions are organized by categories and age groups [2].

Cycling is a physically demanding sport, especially from a metabolic standpoint, given its classification as an endurance activity with substantial aerobic demands [3].

This is demonstrated by the high levels of maximum oxygen consumption and power output at the lactate threshold that have been recorded in competitive road cyclists during laboratory assessments [4]. However, it is crucial to account for anthropometric variables, as these factors also significantly impact performance in this sport [4].

In cycling, there are other factors related to cyclist performance, such as anthropometric, physiological, psychological, and biomechanical variables that influence athletic performance [4,5,6,7,8]. Although it is considered an individual sport, several factors inherent to the discipline affect the competitive outcome. Among these are competitive strategies with the team and those imposed by the characteristics of the terrain [7].

Science is advancing in cycling research, encompassing mechanical, physiological, and nutritional aspects, whose benefits translate into improved performance, which is reflected in competition [7,8]. Nutrition is one of the many factors to consider when planning cyclists’ training and competitions [9]. Within this context, sports supplementation plays an important role in competitive cycling. Thus, the use and optimization of sports supplements (SSs) in relation to the goals established in training or competitions take on special significance in the world of cycling [10]. This planning involves considering the type, timing, and quantity of food intake, along with the simultaneous use of ergogenic aids. These crucial factors can directly and beneficially impact sports performance [8,9,10].

The primary goals of using nutritional supplements (SSs) are to enhance (i) the availability of convenient energy and macronutrients, (ii) direct performance benefits, (iii) indirect support for intense training regimens, and (iv) the management of micronutrient deficiencies [11]. Micronutrients are essential for processes that support sports performance, such as energy production and the creation of new cells and proteins. A clear deficiency in one or more of these nutrients can significantly impair sports performance, either directly or by reducing an athlete’s ability to train effectively (e.g., iron deficiency anemia) or stay healthy and injury-free (e.g., the impact of vitamin D deficiency on bone health). Athletes are susceptible to poor eating habits and increased nutrient loss or requirements, which may make them more prone to deficiencies due to higher nutrient turnover or losses. Additionally, subclinical deficiencies, which are difficult to assess due to a lack of clear metrics or universal thresholds for adequacy, pose a challenge. When suboptimal nutritional status is identified, using nutrient supplements to correct or prevent deficiencies can be a crucial part of the treatment plan [12].

The consumption of SSs has exponentially increased in recent years [11]. Many athletes use these SSs without identifying the potential risks associated, such as the absence of active ingredients, the presence of harmful substances, or doping agents [9,10]. The scientific community defines an SS as a food, food component, nutrient, or non-food component intentionally ingested as part of a regular diet to achieve a specific effect on health or performance [11]. Furthermore, the scientific community classifies SSs in various ways, with one of the most prominent classifications being provided by the Australian Institute of Sport (AIS, 2023), which establishes four groups (A, B, C, and D) based on the degree of scientific evidence [13]. Group A includes SSs related to the improvement of health and performance in athletes, with subgroups comprising medical supplements, performance supplements, and sports foods. Group B includes those SSs that require more research and have moderate use. Group C consists of supplements with no evidence of beneficial effects. Group D encompasses prohibited or contaminant substances whose use can lead to a positive result in a doping test [13].

The use of SSs is widespread among both recreational and elite athletes, with the primary goal of enhancing athletic performance [6,7,14,15,16,17,18,19]. The type of physical activity, level of competition, and sex are factors that influence the consumption of these supplements. Evidence shows an increase in SS consumption among elite competitive athletes compared to those at lower levels in the same sport [18,19]. Although there are a wide range of SSs available, a high percentage of supplements are particularly popular, such as carbohydrate drinks, protein bars, sports gels, caffeine, whey protein, creatine, and branched-chain amino acids (BCAAs) [6,7,15,16,17,18,19]

One aspect that appears to influence the consumption of sports supplements (SSs) is sex [12,17]. It seems that SS consumption is higher in men than in women of the same category, level, and sport [12,17,18]. Despite advancements in gender equality and the increasing participation of women in various sports, there is a notable lack of scientific studies aimed at establishing specific nutritional recommendations for female athletes and other physically active women [20].

Research on collegiate female athletes indicates that over half (65.4%) use either traditional supplements (such as single and multivitamins/minerals) or non-traditional supplements (including herbals, botanicals, and other biologics and nutrients) at least once a month [21]. Recently, a standardized audit of the literature assessed the representation of various recognized sports supplements (such as beta-alanine, caffeine, creatine, glycerol, nitrates/beet juice, and sodium bicarbonate) concerning female athletes [22]. A current review revealed a significant lack of detailed information on supplement usage and dosages specific to female athletes [23]. Understanding the individual patterns and needs of female athletes is necessary given their unique physiological characteristics. Therefore, it is essential to encourage targeted research to address the existing gaps in sports nutrition for women.

Currently, there are various studies on SS consumption in male cyclists [24,25,26,27]. These studies reported varying consumption of supplements among cyclists. Specifically, a prevalence of 100% was found in Canadian Olympic cyclists during the Sydney Olympics [24]; 97.5% in elite U-23 male cyclists [25]; 85% in high-level Spanish cyclists [26]; and 62.5% in federated road cyclists [27]. However, no studies have specifically analyzed the prevalence and consumption patterns among female federated cyclists.

Therefore, the present study aims to analyze sports supplement use patterns among female road cyclists, with a focus on identifying any differences between categories and assessing the current level of evidence supporting the supplements being used.

## 2. Materials and Methods

### 2.1. Type of Study and Sample Size

This quantitative, cross-sectional, descriptive study investigated the use of sports supplements among female road cyclists affiliated with the Andalusian Cycling Federation (FAC). Participants were chosen through non-probabilistic sampling from the FAC, sports associations, and cycling clubs in the Andalusian region. Notably, the study sample comprised 85.18% of the total number of female road cyclists affiliated with the FAC.

### 2.2. Participants

A total of 316 federated women cyclists participated in this study; all were adults with an average age of 39.7 ± 10.4 years. All participants were familiar with training and competition and were free of injuries or health issues at the time of data collection. The classification was based on the Union Cycliste Internationale regulations, with 16 women in the Sub-23 category, 28 in the Cyclotourist category, 54 in the Elite category, 72 in the Master-30 category, 102 in the Master-40 category, and 44 in the Master-50 category. Table 1 provides detailed information on the participants’ ages, height, weight, years of federation membership, weekly training days, daily training hours, and the number of competitions they participated in per season.

### 2.3. Instruments

For data collection in the present study, a questionnaire previously used in similar studies was employed [18,19,27,28]. The questionnaire was previously validated for its content, application, structure, and presentation [29]. The questionnaire employed consisted of three main sections dedicated to anthropometric and personal information (i.e., Section 1), a sports modality practice section which included questions concerning aspects such as category, type, experience, weekly training time, and SS consumption (i.e., Section 2), and a segment focused on factors related to the consumption of SSs (i.e., Section 3). The latter section included information on the types of supplements consumed, reasons for their consumption, sources of advice, places of purchase, times of intake, perceptions of the results obtained, and aspects related to doping. It is important to emphasize that this questionnaire was one of the 57 deemed suitable for accurately assessing athletes’ supplement use out of 164 reviewed by Knapik et al. (2016) in their systematic review and meta-analysis [17].

### 2.4. Procedure

To select participants for the study, clubs and associations registered with the FAC were contacted via email. A link to the form was included, ensuring that detailed information about the procedure was provided to the participants. The study’s characteristics were explained through an introductory letter and explanatory document, and an informed consent document was provided. Data collection was conducted in person at competitions held across Andalusia between January and May 2024. All respondents completed the questionnaire anonymously and voluntarily, always adhering to the principles of the Declaration of Helsinki for research involving humans, with the approval of the ethics committee of Pablo de Olavide University (reference number: 27/7-3).

### 2.5. Statistical Analysis

Kolmogorov–Smirnov and Levene’s tests were used to verify normal distribution and homoscedasticity. Descriptive statistics are reported as means (M) ± standard deviations (SDs) for quantitative variables and as percentages and frequencies for qualitative variables. For inferential analysis, Chi-square tests were used with contingency tables to examine differences between categories. A one-way ANOVA was conducted for pairwise comparisons between categories when assumptions of normality and homogeneity of variance were satisfied; otherwise, a generalized linear model with Bonferroni correction was applied for pairwise comparisons. A significance threshold of *p* < 0.05 was used. All statistical analyses were performed with the Statistical Package for the Social Sciences version 20 for Windows (SPSS) (IBM, Armonk, NY, USA).

## 3. Results

### 3.1. General Aspects of Sport Supplement Consumption

A total of 85.1% of the surveyed cyclists consumed SSs during the season when the data were collected. Represented by categories, 75% of Sub-23, 79.6% of Elites, 91.7% of Master-30, 94.1% of Master-40, 79.5% of Master-50, and 60.7% of Cyclotourists consumed SSs. Significant differences (*p* < 0.05) were found between the Master-30 and Cyclotourist categories, and between the Master-40 and Cyclotourist categories.

### 3.2. Main Purposes of Sport Supplement Consumption

**Figure 1:**
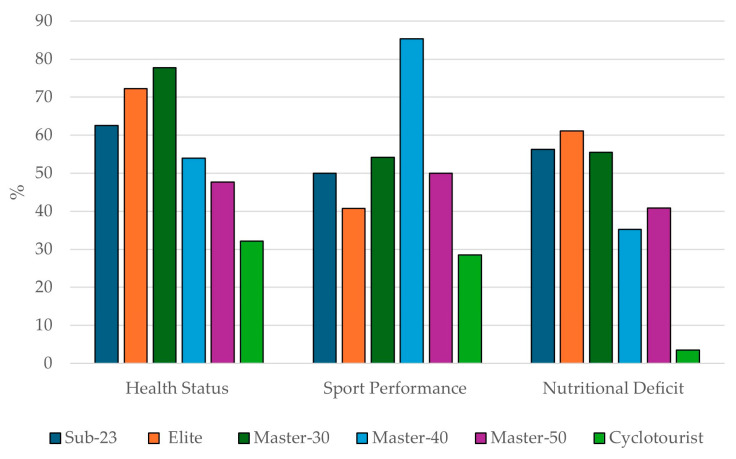
Reasons for sports supplement use by different categories.

The main reasons why the cyclists consumed SSs were health maintenance (60.1%), performance enhancement (58.9%), and addressing dietary deficiencies (43.3%). Figure 1 shows the most chosen reasons for SS use according to the different categories, with Master-30 standing out for health maintenance (77.8%), Master-40 for performance enhancement (85.2%), and Elite for addressing dietary deficiencies (61.1%).

In pairwise comparisons of the most frequent reasons according to the studied categories, significant differences (*p* < 0.05) were observed in the main reasons, with the Cyclotourist category showing the largest number of differences compared to the rest. On the other hand, the S-23 category did not show any significant differences. Similarly, nutritional deficiency was the reason for consumption that presented the largest number of differences in pairwise comparisons by category, while performance enhancement showed the smallest number of differences (Table 2).

### 3.3. Main Purchase Locations for Sports Supplements

**Figure 2:**
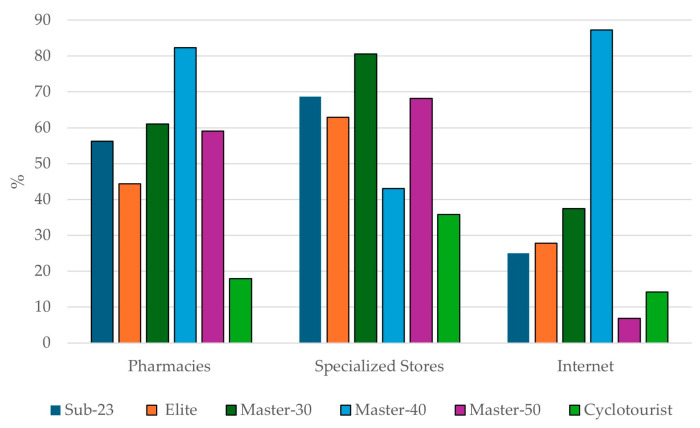
Main places for purchasing sports supplements by different categories.

Pharmacies (60.8%), specialized stores (59.2%), and the Internet (44.9%) were the most frequented places for purchasing SSs by the total sample. Figure 2 shows the preferred places for buying SSs according to the different categories. Notably, the Master-40 category was the group that bought the most from pharmacies (82.4%), the Master-30 category bought the most from specialized stores (80.6%), and the Master-40 category bought the most through the Internet (87.3%).

In pairwise comparisons of the most frequent places according to the studied categories, significant differences (*p* < 0.05) were observed in all comparisons except for specialized stores in the S-23 category compared to the others. Similarly, the Master-40 category showed significant differences compared to all other categories in the purchase of SSs via the Internet, and the Cyclotourist category showed significant differences compared to all other categories in the purchase of SSs from pharmacies (Table 3).

### 3.4. Main Advisors for Sports Supplement Consumption

**Figure 3:**
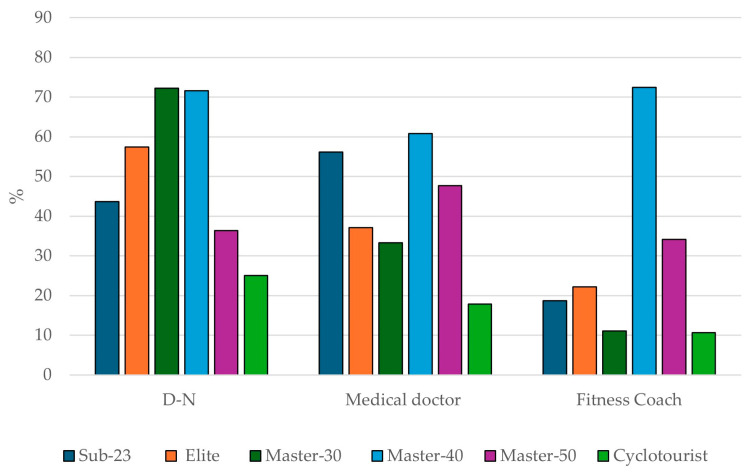
Main sources of advice on sport supplement use by different categories.

Dietitian-nutritionists (D-Ns) (58.9%), medical doctors (44.6%), and fitness coaches (36.4%) were the main advisors on SS consumption. Figure 3 shows the primary sources of advice chosen by the sample for SS consumption, divided by category. Notably, the Master-30 category most frequently consulted D-Ns (72.2%), while the Master-40 category relied most on medical doctors (60.8%) and fitness coaches (72.5%). Conversely, it is important to highlight that the Cyclotourist category consulted D-Ns (25.0%) and medical doctors (17.8%) the least for SS advice.

Pairwise comparisons of the main advisors for SS consumption showed significant differences in at least one pairwise comparison between categories regarding advice from physical trainers. Examining the categories, the Master-40 category had the largest number of differences compared to the other categories for the most frequent advisors (Table 4).

### 3.5. Number of Sports Supplements Consumed

The average number of SSs consumed across the entire sample was 7 ± 6. The Sub-23 category had the highest average consumption, at 9 ± 8 supplements, while the Cyclotourist and Master-50 categories had the lowest, with averages of 4 ± 5 and 5 ± 5 supplements, respectively. Table 5 details the consumption of SSs by groups and subgroups, as categorized by the AIS (2023) based on levels of evidence for both the overall sample and each category.

Among the categories, the Elite group exhibited the most variation in SS consumption across different evidence-based groups (Groups A, B, and C), whereas the Sub-23 category showed the least variation, differing from Cyclotourists only in Group A.

When analyzing the different SS groups and subgroups according to evidence levels (AIS), a greater number of differences were observed in the total number of supplements consumed in Group A. However, no significant differences were found within the subgroups of this category. In contrast, Group D showed no differences between categories, and only four women consumed supplements from this group.

### 3.6. Most Consumed Sports Supplement

**Figure 4:**
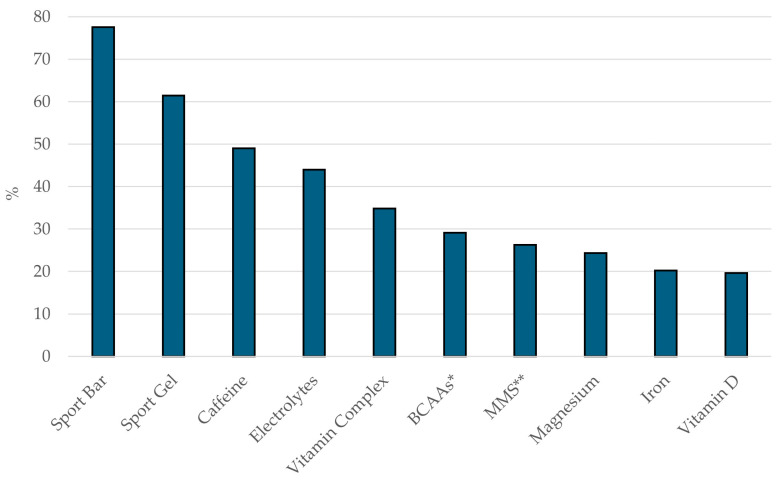
The main supplements consumed by the overall sample. * BCAA: branched-chain amino acids; ** MMS: mixed macronutrient supplement.

Figure 4 shows the top ten SSs consumed by the sample. The five most consumed SSs by the sample were sports bars (77.5%), sports gels (61.4%), caffeine (49.1%), electrolytes (43.9%), and multivitamins (34.8%). Eight out of the ten most consumed SSs belong to the group with the largest level of evidence according to the AIS (Group A). Sports bars, energy gels, electrolytes, and mixed macronutrient supplements belong to the sports foods subgroup; caffeine belongs to the performance supplement subgroup; and multivitamins, iron, and vitamin D belong to the medical supplement subgroup. The other two most consumed SSs, i.e., BCAAs (29.1%) and Magnesium (24.3%), belong to Group C. In the performance supplement subgroup (Group A), most supplements were consumed at low rates, except for caffeine (49.1%). Specifically, beta-alanine was consumed by 10.4%, sodium bicarbonate by 8.2%, creatine by 5.7%, dietary nitrate/beetroot juice by 3.8%, and glycerol by 0% of participants.

As can be observed in Table 6, which displays the most consumed SSs by the total sample and each of the categories, the top three consumed SSs across different categories are in Group A, except for the Master-50 and Cyclotourist categories, which have BCAAs as their first (40.1%) and third (25%) most consumed supplements, respectively.

In pairwise comparisons of the usage percentages of the most consumed SSs across the studied categories, significant differences (*p* < 0.05) were observed for all SSs when comparing the Elite, Master-30, and Master-40 categories against the rest of the categories. In contrast, the Sub-23 category showed the least difference in the usage percentages of the most consumed SSs compared to the other categories. When examining SSs and their usage percentages, sports bars and caffeine supplements showed the largest number of significant differences in pairwise comparisons among the different categories. Conversely, electrolytes, mixed macronutrient supplements, and vitamin D presented the smallest number of significant differences in the percentage of usage in pairwise comparisons across the different categories (Table 6).

## 4. Discussion

This study sought to analyze sports supplement consumption patterns among female road cyclists, with an emphasis on identifying prevalence and consumption trends related to various influencing factors. Although previous research has investigated supplement use among male cyclists [24,25,26,27], there is a notable lack of dedicated studies focusing specifically on female cyclists. This research addresses this gap by examining their consumption patterns and prevalence, considering specific variables, and categorizing them into distinct groups.

### 4.1. General Aspects of Sports Supplement Consumption

A total of 85.1% of the surveyed cyclists consumed SSs. These figures are lower than those reported for Canadian Olympic cyclists (100%) during the Sydney Olympic Games [24] and for elite under-23 male cyclists (97.5%) [25]. Although these data reinforce the idea that the level of competition is a factor that determines SS consumption [17], this is diminished when observing the similarity with the results found in high-level Spanish cyclists (85%) [26], or the lower figures presented by male federated road cyclists of a similar level (62.5%) [27].

Sex and competitive level are two of the variables that most influence SS consumption [17]. In this regard, it is worth noting that the results found in the present study support the hypothesis that there is higher SS consumption with higher levels of training or competition [11,12]. However, they contradict the hypothesis that women consume less SSs than men at a similar competitive level [11,12].

### 4.2. Main Purposes of Sports Supplement Consumption

The reason for SS consumption is addressed in the existing literature, providing a real perspective on its use [12,26,29]. The main reasons for SS consumption in the present study are in line with those reported in a very similar study conducted on male road cyclists [27], where health status (78.2%), athletic performance (67.9%), and nutritional deficiency (47.5%) were the top three choices. These results differ from findings in various studies on other endurance sports involving participants of similar ages (between 34.8 and 43.1 years), where athletic performance was the most frequently chosen reason (from 47.4% to 82.3%) [18,19]. Nutritional deficiency (43.3%) was the third most mentioned reason in the sample of the present study. These results are similar to those of a study conducted with elite under-23 male cyclists, where 49.1% cited “addressing a dietary deficiency” as their main reason [25].

Overall, this research highlights that, among federated female road cyclists, the primary motivations for SS consumption align with those observed in existing studies of federated athletes. Specifically, performance and health emerged as the most common reasons for SS use, consistent with findings reported in the literature [18,19,25,27].

### 4.3. Main Purchase Locations for Sports Supplements

The location where SSs are purchased plays a critical role in ensuring their appropriate use as it often provides better guidance and access to higher-quality products [30,31]. In this study, the primary sources of SSs for the participants were pharmacies (60.8%) and specialized stores (59.2%). These findings are similar to the results reported in a recent study, where pharmacies (62.5%), nutrition stores (58.3%), and the Internet (46.8%) were the primary sources of SS purchases [27]. In the same way, Baltazar-Martins et al. (2022) reported that specialized stores (45%) were the most common site of SS purchase among elite Spanish athletes [26]. These options help minimize the risk of SS contamination with banned substances, which is a concern with online purchases [31,32]. This finding is consistent with the main reason for SS use identified in our study, which was health status. This differs from data on elite Spanish athletes across various sports, where only about 1% reported buying SSs from pharmacies [26]. Similar trends have been observed in other endurance sports disciplines [18,19].

The Internet, chosen by 44.9% of participants, is frequently cited as a preferred venue for purchasing SSs (24.2%) [26]. This trend suggests that a significant proportion of female cyclists might be at risk of inadvertent doping due to their SS purchasing practices [33,34,35]. Although the global SS industry has expanded rapidly, this growth introduces several risks for both elite and recreational athletes [36]. The lack of strict international regulations on dietary supplements, coupled with risks of contamination and insufficient guidance on correct usage and scientific backing, heightens the probability of improper or excessive supplement intake and inadvertent doping, especially in the largely unregulated online marketplace [33]. Research has highlighted concerns regarding online purchases, indicating that supplements acquired through these platforms may contain undisclosed ingredients, incorrect dosages, or contaminants, which could endanger athletes’ health, performance, and careers [36,37,38].

### 4.4. Main Advisors for Sports Supplement Consumption

It is important to highlight the significance of professionals or sources of advice regarding SS consumption. The main sources of advice in this study were D-Ns (58.9%), medical doctors (44.6%), and fitness coaches (36.4%). The findings from this study are quite comparable, though not in the same sequence, to those observed in a recent study on male road cyclists [27] and in research on other endurance sports disciplines [18,19]. However, these findings differ from those reported in a study of elite Spanish athletes, where the majority of supplement users depended on their own judgment to acquire valid and accurate information about supplement efficacy, without consulting any professionals [26]. Similarly, other studies indicate that athletes often rely on less reliable sources of information, such as teammates, friends, family members, or the Internet [25,39,40].

The role of D-Ns is gaining significance, as recent research on supplement consumption highlights them as either the primary or secondary source of advice for athletes [18,19]. Athletes who receive information from a dietitian-nutritionist as their primary source of nutritional advice have better eating habits, a better understanding of nutrient timing, and a stronger scientific basis for SS selection [41].

When comparing categories, the Cyclotourist group was found to be the least likely to seek advice on SSs from D-Ns (25.0%), and medical doctors (17.8%). This highlights the importance of relying on credible sources for SS guidance, as inadequate or incorrect advice can result in the use of supplements lacking scientific support or, more critically, lead to unintentional doping and health risks [37,38]. Additionally, it supports the hypothesis of potentially poorer SS use among amateurs or lower-level athletes [17,32].

### 4.5. Number of Sports Supplements Consumed

An average of 7 ± 6 SSs was consumed by the cyclists. These results differ significantly from those reported in a study on male cyclists (12 SSs) [22]. However, they are similar to those in other endurance sports such as mountain running (7 SSs) and triathlon (8 SSs) [18]. As previously mentioned, gender is one of the variables that most influence SS consumption [11,12,17]. In this regard, although the prevalence of consumption did not support this aspect, the number of SSs consumed does support the hypothesis that women consume fewer SSs than men at a similar competitive level [12,17].

The level of competition is another relevant aspect of SS consumption [12,17]. Thus, it can be observed that the category with the lowest level of competition, Cyclotourist, consumed the fewest SSs in the entire sample, once again supporting the hypothesis that higher levels of training or competition are associated with greater SS consumption [12,17].

Regarding the type of SSs and its level of evidence according to the AIS [13], the high consumption of Group A SSs compared to other groups stands out. However, this difference is smaller in categories with lower competitive levels (Master-50 and Cyclotourist). These results are linked to factors such as the place of purchase and the source of information, which is a very positive finding. This trend is supported by recent similar studies where Group A SSs were the most frequently consumed [18,27,28]. Scientific dissemination about SSs is positively impacting the target population. Additionally, evidence suggests that educational interventions in nutrition can improve athletes’ knowledge of sports nutrition [32], making it potentially useful.

Within Group A, there was a notable difference in the amount of SSs used in the sports food subgroup compared to the other two subgroups. These results are in agreement with data from recent studies in endurance sports [18,19]. The characteristics of training, competitions, and weather conditions in cycling justified the use of SSs from the sports foods subgroup (Group A), such as sports gels, sports drinks, sports bars, isolated proteins, mixed macronutrient supplements, and electrolyte supplements [13]. These factors are crucial in cycling as they assist in managing performance-limiting variables and mitigating health risks [6,42]. They play a key role in maintaining hydroelectrolytic balance, preventing dehydration, and supporting endurance, strength, blood volume, and cognitive function. Additionally, they aid in replenishing muscle glycogen, minimizing muscle damage, and enhancing recovery after exercise [7,8,10,43].

Although to a lesser extent, it is significant that Group C, which included supplements with evidence against their effectiveness [13], was the second most consumed group. This finding is consistent with recent research where Group C was also the second most frequently used after Group A [18,19,27]. The use of these supplements can potentially lead to negative outcomes such as positive doping tests, decreased performance, and adverse health effects [31]. Competitive athletes should be cautious of overuse and potential negative interactions from combining multiple supplements [44], as well as the risk of inadvertent doping due to insufficient quality control in some Group C supplements [45]. Better-informed athletes are likely to use fewer supplements promoted by the industry [26,36,46].

### 4.6. Most Consumed Sports Supplements

The findings indicate that eight of the ten most frequently used supplements by the sample fall into the category with the highest level of evidence as defined by the AIS (Group A): four to the sport foods subgroup, three to the medical supplement subgroup, and one to the performance supplement subgroup. Although this is a commendable aspect, it is crucial to recognize that several scientifically validated supplements, including beta-alanine, sodium bicarbonate, creatine, dietary nitrate/beetroot juice, and glycerol, are not among the top twenty most frequently consumed supplements.

Sport bars (77.5%) and gels (61.4%) were the most consumed SSs. This is consistent with results from similar research on endurance athletes, where sports drinks, energy bars, and sports gels were identified as the three most commonly consumed supplements [18,19,27]. Dietary supplements offer practical support for meeting energy and nutrient needs across various sports contexts [11,12]. These supplements, often marketed as sport drinks, bars or gels, carbohydrate (CHO) and protein supplements, liquid meals, or electrolyte replacements, are particularly relevant for female athletes [20,23]. CHO supplementation, such as in sports drinks, bars and gels, is commonly used by women athletes to stabilize blood glucose levels and delay glycogen depletion during exercise [11,12,20].

Mixed macronutrient supplement (26.3%) was other of the supplements most used by the sample. While CHO drinks are popular, combining CHO with protein has been shown to enhance endurance performance more effectively than CHO alone [20]. In a study involving trained female cyclists, a mixed CHO-protein supplement was found to be more effective than a traditional CHO-only supplement in increasing time to exhaustion. This effect may be attributed to protein’s role in boosting insulin production, enhancing glucose clearance, and potentially reducing exercise-induced muscle damage [47].

Caffeine supplements (61.4%) were the third of the most used SSs by the participants. Despite this, only a fraction (13%) of caffeine studies have focused specifically on women. Caffeine supplementation has been shown to improve various performance metrics, including velocity and power during strength training, muscular endurance, and performance in sprint and agility tests [20]. The ergogenic effects of caffeine are well documented, enhancing both endurance and high-intensity efforts by increasing alertness, mood, and cognitive performance, as well as improving muscle coordination and calcium bioavailability [23]. Furthermore, this highlights the importance of incorporating these scientifically validated supplements into training regimens, particularly for women in sports like cycling, where optimal performance is crucial.

Beta-alanine and sodium bicarbonate (NaHCO_3_^−^) function as buffering agents [48,49]. The efficacy of NaHCO_3_^−^ is due to its enhancement of both extracellular and intracellular buffering capacity, which raises blood bicarbonate levels and pH. Beta-alanine, on the other hand, works by increasing muscle carnosine concentration, providing an ergogenic benefit during high-intensity exercise [50]. Although these supplements have robust scientific backing for their effectiveness, their use among women remains relatively low [28,51,52], aligning with our findings, where beta-alanine (10.4%) and NaHCO_3_^−^ (8.2%) were two of the SSs underused by the sample, although both are in the Group A.

Despite the low usage of creatine in the present study (5.7%), it is one of the most widely used ergogenic aids among athletes [17]. Studies have shown that its supplementation increases intramuscular creatine concentrations, enhancing performance in high-intensity exercises and training adaptations [53]. Additionally, creatine aids post-exercise recovery, injury prevention, thermoregulation, rehabilitation, and neuroprotection in cases of concussions and spinal cord injuries [54]. Given its ability to improve anaerobic work capacity and repeated peak performance, creatine supplementation can be particularly beneficial for sports such as mountain biking, road cycling, and triathlons [55].

Although nitrate supplementation is gaining popularity as a sports nutrition supplement [13], it remains underused [17,18,19,26,27]. This is reflected in our study, where only 3.8% of participants used nitrate supplements. The nitric oxide generated by these supplements enhances vasodilation, blood circulation, and muscle contraction strength [56,57]. Beetroot juice, a nitrate-rich nutritional source, can increase the bioavailability of nitric oxide, improving muscle strength performance, endurance capacity, and high-intensity intermittent efforts [56,57,58]. Additionally, nitrate supplementation in cycling can be beneficial as it reduces the oxygen cost of submaximal exercise and improves exercise tolerance and performance [58].

A recent systematic review highlighted strong evidence supporting the ergogenic benefits of certain supplements, including caffeine, creatine, alkalizing agents, and nitrates. However, more research is necessary to optimize their application and understand their specific advantages in female athletes [20].

Interestingly, iron was one of the ten most consumed supplements by the sample (20.3%). Iron-deficiency anemia and iron deficiency are five to seven times more common in women than in men [59]. These prevalence rates are related to sex differences [60], poor dietary intake, exercise-induced iron losses, and disruptions in iron regulation during the menstrual cycle [61,62,63]. This is an important finding, as iron presents itself as an effective supplement for female athletes [20,23].

Given the narrow margin between success and defeat in sports, many athletes seek to enhance their performance through the use of SSs [64]. Although supplements should be a complementary part of an athlete’s proper planning, which includes factors like genetics, training, and diet, there tends to be an excessive emphasis on their consumption [32]. Despite the widespread and standardized use of SS, it is crucial for both health professionals and athletes to conduct a cost–benefit analysis before their use, considering their safety, efficacy, and legality [12,31]. Moreover, the consumption of supplements should complement sports planning and should not replace an adequate diet or proper food choices [32,65].

### 4.7. Limitations and Future Perspectives

Although our study provides new insights and involves a substantial sample of federated female road cyclists, several limitations must be noted. A key limitation is the reliance on self-reported data, which could introduce inaccuracies. Additionally, the study focused exclusively on supplements used during the specific season of data collection, potentially overlooking long-term trends in supplement use and their effects on athletes’ health. Moreover, despite including examples in the questionnaire to assist in supplement identification, there remains a chance that some cyclists may have misreported their actual supplement use. These limitations highlight the need for careful interpretation of the study’s findings.

Standardizing and categorizing SSs are crucial for effective management among both amateur and elite athletes. Addressing the risks associated with SS use, evaluating their effectiveness, and optimizing nutrient intake from food to reduce reliance on supplements are important areas for ongoing research and education. Given the low consumption of many supplements in the performance supplement subgroup, such as beta-alanine, sodium bicarbonate, creatine, dietary nitrate/beetroot juice, and glycerol, it is essential to inform athletes about their potential benefits. Additionally, studying their specific ergogenic effects through randomized controlled trials in the context of women’s road cycling is recommended.

## 5. Conclusions

The prevalence of sports supplement use among the female road cyclists in this study is notably high, at 85.1%. The main places of purchase and the primary advisors for SS use, pharmacies (60.8%) and dietitian-nutritionists (58.9%), respectively, are reliable. The most frequently consumed SSs, sports bars (77.5%), sports gels (61.4%), and caffeine (49.1%), are supported by scientific evidence. Remarkably, 80% of the top ten most frequently consumed supplements are in the category with the highest level of evidence (Group A), with participants using an average of 5 ± 3 supplements each. The use of Group D supplements or doping substances is practically non-existent. These results provide the first evidence of SS consumption among female federated road cyclists and highlight specific patterns to consider.

## Figures and Tables

**Table 1:** Demographic and training characteristics of participants.

Category	Age (Years)	Height (cm)	Weight (kg)	Years Licensed	Weekly Training Days	Daily Training Hours	No. Competitions in the Season
Total (*n* = 316)	39.7 ± 10.4	162.8 ± 7.0	59.8 ± 7.2	7.3 ± 3.2	4.3 ± 1.1	2.5 ± 0.7	9 ± 3
Sub-23 (*n* = 16)	20.6 ± 1.8	164.8 ± 5.5	57.8 ± 5.8	2.3 ± 1.3	4.7 ± 0.6	2.5 ± 0.5	9 ± 4
Elite (*n* = 54)	26.7 ± 2.0	162.7 ± 7.4	58.5 ± 7.0	7.4 ± 2.7	4.4 ± 1.1	2.4 ± 0.7	10 ± 3
Master-30 (*n* = 72)	35.1 ± 2.6	163.6 ± 7.5	59.7 ± 6.8	4.1 ± 2.4	3.9 ± 1.4	2.5 ± 0.6	11 ± 3
Master-40 (*n* = 102)	44.7 ± 2.8	160.6 ± 6.8	58.0 ± 6.7	9.0 ± 2.5	4.4 ± 0.8	2.5 ± 0.6	9 ± 2
Master-50 (*n* = 44)	54.7 ± 3.6	165.7 ± 4.0	66.6 ± 5.8	8.2 ± 2.8	4.0 ± 1.1	2.3 ± 0.6	6 ± 2
Cyclotourist (*n* = 28)	46.1 ± 8.7	163.5 ± 7.6	59.8 ± 7.3	5.4 ± 3.9	4.6 ± 1.2	2.8 ± 1.3	5 ± 3

Data are presented as the M ± SD. The categories are distributed by the Union Cycliste Internationale (UCI).

**Table 2:** Main consumer purposes for sports supplement consumption by the overall sample and each category.

	Total	Sub-23	Elite	Master-30	Master-40	Master-50	Cyclotourist
	(*n* = 316)	(*n* = 16)	(*n* = 54)	(*n* = 72)	(*n* = 102)	(*n* = 44)	(*n* = 28)
Health Status	60.1	62.5	72.2 ^a^	77.8 ^bcd^	53.9 ^b^	47.7 ^c^	32.1 ^ad^
Sport Performance	58.9	50.0	40. 7 ^a^	54.2 ^b^	85.3 ^abc^	50.0	28.6 ^c^
Nutritional Deficit	43.4	56.2	61.1 ^ab^	55.6 ^c^	35.3 ^ad^	40.9 ^e^	3.6 ^bcde^

Data are presented as the %. Differences between categories are displayed using a generalized linear model. The same superscript (letters) indicates differences in pairs (Bonferroni *p* < 0.05).

**Table 3:** The most common places for purchasing sports supplements by the overall sample and each category.

	Total	Sub-23	Elite	Master-30	Master-40	Master-50	Cyclotourist
	(*n* = 316)	(*n* = 16)	(*n* = 54)	(*n* = 72)	(*n* = 102)	(*n* = 44)	(*n* = 28)
Pharmacies	60.8	56.2 ^a^	44.4 ^bc^	61.1 ^d^	82.3 ^be^	59.1 ^f^	17.9 ^acdef^
Specialized stores	59.2	68.7	62.9 ^a^	80.6 ^bc^	43.1 ^b^	68.2 ^d^	35.77 ^acd^
Internet	44.9	25 ^a^	27.8 ^bc^	37.5 ^def^	87.3 ^abdgh^	6.8 ^ceg^	14.23 ^fh^

Data are presented as the %. Differences between categories are presented using a generalized linear model. The same superscript (letters) indicates differences in pairs (Bonferroni *p* < 0.05).

**Table 4:** Main sources/advisors of information on sports supplement use by the overall sample and each category.

	Total	Sub-23	Elite	Master-30	Master-40	Master-50	Cyclotourist
	(*n* = 316)	(*n* = 16)	(*n* = 54)	(*n* = 72)	(*n* = 102)	(*n* = 44)	(*n* = 28)
D-N	58.9	43.7	57.4 ^a^	72.2 ^bc^	71.6 ^de^	36.4 ^bd^	25.0 ^ace^
Medical doctor	44.6	56.2	37.1	33. 3 ^a^	60.8 ^ab^	47.7 ^c^	17.8 ^bc^
Fitness coach	36.4	18.7 ^a^	22.2 ^b^	11.1 ^cd^	72.5 ^abcef^	34.1 ^de^	10.7 ^f^

Data are presented as the %. Differences between categories are presented using a generalized linear model. The same superscript (letters) indicates differences in pairs (Bonferroni *p* < 0.005). D-N: dietitian-nutritionist.

**Table 5:** The number of sports supplements used by the overall sample and each category, organized by supplement groups based on AIS (2023) evidence levels [13].

SS Group by Level of Evidence (AIS)		Total (*n* = 316)	Sub-23 (*n* = 16)	Elite (*n* = 54)	Master-30 (*n* = 72)	Master-40 (*n* = 102)	Master-50 (*n* = 44)	Cyclotourist (*n* = 28)
**Group A**	Sport Food	3 ± 3	3 ± 2	3 ± 2	4 ± 2	3 ± 1	2 ± 1.	2 ± 2
	Medical Supplement	1 ± 1	1 ± 1	1 ± 0.8	1 ± 1	1 ± 1	1 ± 2	0 ± 1
	Performance Supplement	1 ± 1	1 ± 1	1 ± 1	1 ± 1	1 ± 1	0 ± 0	0 ± 1
	Total Group A	5 ± 3	5 ± 4 ^a^	5 ± 3 ^bc^	6 ± 4 ^def^	4 ± 2 ^dg^	3 ± 3 ^be^	3 ± 3 ^acfg^
**Group B**		0 ± 1	1 ± 1	0 ± 0 ^ab^	1 ± 1 ^a^	1 ± 1 ^b^	0 ± 1	1 ± 1
**Group C**		2 ± 3	3 ± 4	1 ± 1 ^a^	1 ± 2	2 ± 4 ^a^	1 ± 2	1 ± 2
**Group D**		0	0	0	0	0	0	0
**Total Supplements**		7 ± 6	9 ± 8	6 ± 4	8 ± 6	7 ± 7	5 ± 5	4 ± 5

Data are presented as the M ± SD. Differences between categories are presented using a generalized linear model. The same superscript (letters) indicates differences in pairs (Bonferroni *p* < 0.005).

**Table 6:** The main supplements consumed by the overall sample and each category.

	Total (*n* = 316)	Sub-23 (*n* = 16)	Elite (*n* = 54)	Master-30 (*n* = 72)	Master-40 (*n* = 102)	Master-50 (*n* = 44)	Cyclotourist (*n* = 28)
Sports bar	77.5	75.0 ^a^	83.3 ^bc^	90.2 ^de^	91.1 ^fg^	36.3 ^abdf^	46.4 ^ceg^
Sport gel	61.3	62.5 ^a^	74.1 ^b^	79.1 ^cd^	61.7 ^ef^	36.3 ^bce^	21.4 ^abdf^
Caffeine	49.1	75 ^a^	48.1 ^b^	77.7 ^cde^	50 ^cfg^	36.3 ^abdf^	25 ^eg^
Electrolytes	43.9	37.5	57.4 ^a^	62.5 ^bc^	32.3 ^b^	36.3	25 ^ac^
Vitamin complex	34.8	31.2	61.1 ^ab^	51.3 ^cd^	14.7 ^ac^	34.1	14.3 ^bd^
BCAA *	29.1	43.7	44.4 ^abc^	47.2 ^def^	15.7 ^ad^	13.6 ^be^	14.3 ^cf^
MMS **	26.2	31.2	31.5 ^a^	56.9 ^bc^	0 ^abd^	31.8 ^d^	10.7 ^c^
Magnesium	24.3	31.2	0 ^abcd^	22.2 ^a^	28.4 ^b^	40.1 ^c^	25 ^d^
Iron	20.2	31.2	40.7 ^a^	16.7 ^b^	17.6 ^c^	15.9 ^d^	0 ^abcd^
Vitamin D	19.6	31.2	0 ^abc^	15.2 ^a^	24.5 ^b^	38.6 ^c^	14.3

Data are presented as the %. Differences between categories are presented using a generalized linear model. The same superscript (letters) indicates differences in pairs (Bonferroni *p* < 0.005). * BCAA: branched-chain amino acids; ** MMS: mixed macronutrient supplement.

## Data Availability

Data are available at the research data repository of the University of Seville—awaiting DOI assignment.

## References

[1] Real Federación Espanola de Ciclismo. https://rfec.com/index.php/es/smartweb/seccion/seccion/rfec/home.

[2] Union Cycliste Internationale. https://www.uci.org/.

[3] Larson D.J., Maxcy J.G. (2015). Human capital development in professional cycling. The Economics of Professional Road Cycling.

[4] Mujika I., Padilla S. (2001). Physiological and performance characteristics of male professional road cyclists. Sports Med..

[5] Atkinson G., Davison R., Jeukendrup A., Passfield L. (2003). Science and cycling: Current knowledge and future directions for research. J. Sports Sci..

[6] Faria E.W., Parker D.L., Faria I.E. (2005). The science of cycling: Factors affecting performance—Part 2. Sports Med..

[7] Santalla A., Earnest C.P., Marroyo J.A., Lucia A. (2012). The Tour de France: An updated physiological review. Int. J. Sports Physiol. Perform..

[8] Phillips K.E., Hopkins W.G. (2020). Determinants of Cycling Performance: A Review of the Dimensions and Features Regulating Performance in Elite Cycling Competitions. Sports Med. Open.

[9] Moro T., Tinsley G., Longo G., Grigoletto D., Bianco A., Ferraris C., Guglielmetti M., Veneto A., Tagliabue A., Marcolin G. (2020). Time-Restricted Eating Effects on Performance, Immune Function, and Body Composition in Elite Cyclists: A Randomized Controlled Trial. J. Int. Soc. Sports Nutr..

[10] Burke L.M. (2001). Nutritional practices of male and female endurance cyclists. Sports Med..

[11] Maughan R.J., Burke L.M., Dvorak J., Larson-Meyer D.E., Peeling P., Phillips S.M., Rawson E.S., Walsh N.P., Garthe I., Geyer H. (2018). IOC consensus statement: Dietary supplements and the high-performance athlete. Br. J. Sports Med..

[12] Maughan R.J., Burke L.M., Dvorak J., Larson-Meyer D.E., Peeling P., Phillips S.M., Rawson E.S., Walsh N.P., Garthe I., Geyer H. (2018). IOC consensus statement: Dietary supplements and the high-performance athlete. Int. J. Sport Nutr. Exerc. Metab..

[13] Australian Institute of Sport Sports Supplement Framework. https://www.ais.gov.au/nutrition/supplements.

[14] Domínguez R., Naderi A., Sánchez-Oliver A.J., Muñoz-López A., Taiar R., Sañudo B. (2022). Supplementation and Ergogenic Aids for Enhancing Muscular Strength Production. Resistance Training Methods.

[15] Burke L.M. (2017). Practical Issues in Evidence-Based Use of Performance Supplements: Supplement Interactions, Repeated Use and Individual Responses. Sports Med..

[16] Burke L.M., Jeukendrup A.E., Jones A.M., Mooses M. (2019). Contemporary Nutrition Strategies to Optimize Performance in Distance Runners and Race Walkers. Int. J. Sport Nutr. Exerc. Metab..

[17] Knapik J.J., Steelman R.A., Hoedebecke S.S., Austin K.G., Farina E.K., Lieberman H.R. (2016). Prevalence of Dietary Supplement Use by Athletes: Systematic Review and Meta-Analysis. Sports Med..

[18] Jiménez-Alfageme R., Martínez-Sanz J.M., Romero-García D., Giménez-Monzo D., Hernández Aparicio S., Sanchez-Oliver A.J., Sospedra I. (2023). Do Spanish Triathletes Consume Sports Supplements According to Scientific Evidence? An Analysis of the Consumption Pattern According to Sex and Level of Competition. Nutrients.

[19] Jiménez-Alfageme R., Domínguez R., Sanchez-Oliver A.J., Tapia-Castillo P., Martínez-Sanz J.M., Sospedra I. (2022). Analysis of the Consumption of Sports Supplements in OpenWater Swimmers According to the Competitive Level. Nutrients.

[20] Larrosa M., Gil-Izquierdo A., Gonzalez-Rodríguez L.A., Muñoz Alferez M.J., San Juan A., Sánchez-Gómez A., Calvo-Ayuso N., Ramos-Alvarez J.J., Fernández-Lázaro D., López-Grueso R. (2023). Nutritional strategies for optimizing health, sports performance, and recovery for female athletes and other physically active women: A systematic review. Nutr. Rev..

[21] Herbold N.H., Visconti B.K., Frates S., Bandini L. (2004). Traditional and Nontraditional Supplement Use by Collegiate Female Varsity Athletes. Int. J. Sport Nutr. Exerc. Metab..

[22] Smith E.S., McKay A.K.A., Kuikman M., Ackerman K.E., Harris R., Elliott-Sale K.J., Stellingwerff T., Burke L.M. (2022). Auditing the Representation of Female Versus Male Athletes in Sports Science and Sports Medicine Research: Evidence-Based Performance Supplements. Nutrients.

[23] Sims S.T., Kerksick C.M., Smith-Ryan A.E., Janse de Jonge X.A.K., Hirsch K.R., Arent S.M., Hewlings S.J., Kleiner S.M., Bustillo E., Tartar J.L. (2023). International Society of Sports Nutrition Position Stand: Nutritional Concerns of the Female Athlete. J. Int. Soc. Sports Nutr..

[24] Huang S.-H., Johnson K., Pipe A.L. (2006). The Use of Dietary Supplements and Medications by Canadian Athletes at the Atlanta and Sydney Olympic Games. Clin. J. Sport Med..

[25] Loraschi A., Galli N., Cosentino M. (2014). Dietary Supplement and Drug Use and Doping Knowledge and Attitudes in Italian Young Elite Cyclists. Clin. J. Sport Med..

[26] Baltazar-Martins G., Brito de Souza D., Aguilar-Navarro M., Muñoz-Guerra J., Plata M.D.M., Del Coso J. (2019). Prevalence and Patterns of Dietary Supplement Use in Elite Spanish Athletes. J. Int. Soc. Sports Nutr..

[27] García-Durán J., González-Jurado J.A., Sánchez-Oliver A.J. (2024). Analysis of Sports Supplement Consumption in 1688 Federated Road Cyclists. Nutrients.

[28] Günalan E., Çavak B.Y., Turhan S., Cebioğlu İ.K., Domínguez R., Sánchez-Oliver A.J. (2022). Dietary Supplement Use of Turkish Footballers: Differences by Sex and Competition Level. Nutrients.

[29] Sánchez-Oliver A.J. (2012). Suplementación Nutricional en la Actividad Físico-Deportiva. Análisis de la Calidad del Suplemento Proteico Consumido.

[30] Petroczi A., Naughton D.P. (2008). The Age-Gender-Status Profile of High Performing Athletes in the UK Taking Nutritional Supplements: Lessons for the Future. J. Int. Soc. Sports Nutr..

[31] Maughan R.J., Shirreffs S.M., Vernec A. (2018). Making Decisions About Supplement Use. Int. J. Sport Nutr. Exerc. Metab..

[32] Mata-Ordonez F., Sánchez-Oliver A.J., Domínguez Herrera R., Villegas García J.A. (2018). Suplementación en el deporte: Directrices desde la responsabilidad profesional. Habilid. Mot..

[33] Martínez Sanz J.M., Ripoll M., Puya-Braza J.M., Segura A., Sánchez-Oliver A., Mata F., Cortell-Tormo J. (2021). Fraud in Nutritional Supplements for Athletes: A Narrative Review. Nutr. Hosp..

[34] Chapple C.I., Russell C.G., Burnett A.J., Woods J.L. (2023). Sports Foods Are Not All They Shake Up to Be: An Audit of Formulated Supplementary Sports Food Products and Packaging in Australian Retail Environments. Front. Nutr..

[35] Geyer H., Parr M.K., Koehler K., Mareck U., Schänzer W., Thevis M. (2008). Nutritional Supplements Cross-Contaminated and Faked with Doping Substances. J. Mass Spectrom..

[36] Garthe I., Ramsbottom R. (2020). Elite Athletes: A Rationale for the Use of Dietary Supplements: A Practical Approach. PharmaNutrition.

[37] Garthe I., Maughan R.J. (2018). Athletes and Supplements: Prevalence and Perspectives. Int. J. Sport Nutr. Exerc. Metab..

[38] Kozhuharov V.R., Ivanov K., Ivanova S. (2022). Dietary Supplements as a Source of Unintentional Doping. BioMed Res. Int..

[39] Froiland K., Koszewski W., Hingst J., Kopecky L. (2004). Nutritional Supplement Use Among College Athletes and Their Sources of Information. Int. J. Sport Nutr. Exerc. Metab..

[40] Petróczi A., Naughton D.P., Mazanov J., Holloway A., Bingham J. (2007). Performance Enhancement with Supplements: Incongruence Between Rationale and Practice. J. Int. Soc. Sports Nutr..

[41] Hull M.V., Jagim A.R., Oliver J.M., Greenwood M., Busteed D.R., Jones M.T. (2016). Gender Differences and Access to a Sports Dietitian Influence Dietary Habits of Collegiate Athletes. J. Int. Soc. Sports Nutr..

[42] Faria E.W., Parker D.L., Faria I.E. (2005). The Science of Cycling: Physiology and Training—Part 1. Sports Med..

[43] Mata F., Grimaldi-Puyana M., Sánchez-Oliver A.J. (2019). Reposición del Glucógeno Muscular en la Recuperación del Deportista. Sport TK Rev. EuroAmer. Cienc. Deporte.

[44] Suzić Lazic J., Dikić N., Radivojević N., Mazić S., Radovanović D., Mitrović N., Lazić M., Zivanic S., Suzić S. (2011). Dietary Supplements and Medications in Elite Sport—Polypharmacy or Real Need?. Scand. J. Med. Sci. Sports.

[45] Outram S., Stewart B. (2015). Doping Through Supplement Use: A Review of the Available Empirical Data. Int. J. Sport Nutr. Exerc. Metab..

[46] Gabriels G., Lambert M. (2013). Nutritional Supplement Products: Does the Label Information Influence Purchasing Decisions for the Physically Active?. Nutrients.

[47] McCleave E.L., Ferguson-Stegall L., Ding Z., Doerner P.G., Wang B., Kammer L.M., Ivy J.L. (2011). A Low Carbohydrate–Protein Supplement Improves Endurance Performance in Female Athletes. J. Strength Cond. Res..

[48] Saunders B., Elliott-Sale K., Artioli G.G., Swinton P.A., Dolan E., Roschel H., Sale C., Gualano B. (2017). β-Alanine Supplementation to Improve Exercise Capacity and Performance: A Systematic Review and Meta-Analysis. Br. J. Sports Med..

[49] Peart D.J., Siegler J.C., Vince R.V. (2012). Practical Recommendations for Coaches and Athletes: A Meta-Analysis of Sodium Bicarbonate Use for Athletic Performance. J. Strength Cond. Res..

[50] Lancha Junior A.H., Painelli V.D.S., Saunders B., Artioli G.G. (2015). Nutritional Strategies to Modulate Intracellular and Extracellular Buffering Capacity During High-Intensity Exercise. Sports Med..

[51] Ventura Comes A., Sánchez-Oliver A.J., Martínez-Sanz J.M., Domínguez R. (2018). Analysis of Nutritional Supplements Consumption by Squash Players. Nutrients.

[52] Moreno B., Veiga S., Sánchez-Oliver A.J., Domínguez R., Morencos E. (2022). Analysis of Sport Supplement Consumption by Competitive Swimmers According to Sex and Competitive Level. Nutrients.

[53] Valenzuela P.L., Morales J.S., Emanuele E., Pareja-Galeano H., Lucia A. (2019). Supplements with Purported Effects on Muscle Mass and Strength. Eur. J. Nutr..

[54] Kreider R.B., Kalman D.S., Antonio J., Ziegenfuss T.N., Wildman R., Collins R., Candow D.G., Kleiner S.M., Almada A.L., Lopez H.L. (2017). International Society of Sports Nutrition Position Stand: Safety and Efficacy of Creatine Supplementation in Exercise, Sport, and Medicine. J. Int. Soc. Sports Nutr..

[55] Forbes S.C., Candow D.G., Neto J.H.F., Kennedy M.D., Forbes J.L., Machado M., Bustillo E., Gomez-Lopez J., Zapata A., Antonio J. (2023). Creatine Supplementation and Endurance Performance: Surges and Sprints to Win the Race. J. Int. Soc. Sports Nutr..

[56] Tan R., Cano L., Lago-Rodríguez Á., Domínguez R. (2022). The Effects of Dietary Nitrate Supplementation on Explosive Exercise Performance: A Systematic Review. Int. J. Environ. Res. Public Health.

[57] Domínguez R., Maté-Muñoz J.L., Cuenca E., García-Fernández P., Mata-Ordoñez F., Lozano-Estevan M.C., Veiga-Herreros P., da Silva S.F., Garnacho-Castaño M.V. (2018). Effects of Beetroot Juice Supplementation on Intermittent High-Intensity Exercise Efforts. J. Int. Soc. Sports Nutr..

[58] Jones A.M. (2014). Influence of Dietary Nitrate on the Physiological Determinants of Exercise Performance: A Critical Review. Appl. Physiol. Nutr. Metab..

[59] DellaValle D.M., Haas J.D. (2011). Impact of Iron Depletion without Anemia on Performance in Trained Endurance Athletes at the Beginning of a Training Season: A Study of Female Collegiate Rowers. Int. J. Sport Nutr. Exerc. Metab..

[60] Murphy W.G., Tong E., Murphy C. (2010). Why Do Women Have Similar Erythropoietin Levels to Men but Lower Hemoglobin Levels?. Blood.

[61] Sim M., Dawson B., Landers G., Trinder D., Peeling P. (2014). Iron Regulation in Athletes: Exploring the Menstrual Cycle and Effects of Different Exercise Modalities on Hepcidin Production. Int. J. Sport Nutr. Exerc. Metab..

[62] Peeling P., Dawson B., Goodman C., Landers G., Wiegerinck E.T., Swinkels D.W., Trinder D. (2009). Training Surface and Intensity: Inflammation, Hemolysis, and Hepcidin Expression. Med. Sci. Sports Exerc..

[63] Peeling P., Dawson B., Goodman C., Landers G., Wiegerinck E.T., Swinkels D.W., Trinder D. (2009). Cumulative Effects of Consecutive Running Sessions on Hemolysis, Inflammation and Hepcidin Activity. Eur. J. Appl. Physiol..

[64] López-González L.M., Sánchez-Oliver A.J., Mata F., Jodra P., Antonio J., Domínguez R. (2018). Acute Caffeine Supplementation in Combat Sports: A Systematic Review. J. Int. Soc. Sports Nutr..

[65] Maughan R.J., Depiesse F., Geyer H., International Association of Athletics Federations (2007). The Use of Dietary Supplements by Athletes. J. Sports Sci..

